# Genetic Diversity of Porcine Epidemic Diarrhea Virus With a Naturally Occurring Truncated ORF3 Gene Found in Guangxi, China

**DOI:** 10.3389/fvets.2020.00435

**Published:** 2020-07-24

**Authors:** Ying Lu, Xueli Su, Chen Du, Liyuan Mo, Purui Ke, Ruomu Wang, Lian Zhong, Cui Yang, Ying Chen, Zuzhang Wei, Weijian Huang, Yuying Liao, Kang Ouyang

**Affiliations:** ^1^Laboratory of Animal Infectious Disease and Molecular Immunology, College of Animal Science and Technology, Guangxi University, Nanning, China; ^2^Laboratory of Poultry, Guangxi Institute of Animal Science, Nanning, China

**Keywords:** pigs, PEDV, ORF3, genetic diversity, naturally truncated gene

## Abstract

Porcine epidemic diarrhea virus (PEDV) is one of the major enteric pathogens, causing severe enteric disease, resulting in enormous economic losses. The ORF3 gene encodes an accessory protein which is related to the infectivity and virulence of PEDV. In this study, 33 PEDV positive field samples were collected from Guangxi, from 2017 to 2019, and the genetic diversity of ORF3 was investigated. Thirty-eight strains of ORF3 were obtained, and these were composed of five strains of ORF3 named Guangxi naturally truncated strains that were 293 bp in length, with continuous deletions from 172 to 554 bp. The Guangxi naturally truncated strains encoded a truncated protein of 89 amino acids, which had clustered into a new group referred to as Group 3, and these might be involved in the variations of virulence. Three genotypes (G1-1 subgroup, G1-3 subgroup, and Group 3) existed simultaneously in Guangxi based on the genetic and evolutionary analysis of the ORF3 gene. The sequence information in the current study will hopefully facilitate the establishment of a diagnostic method that can differentiate the PEDV field stains. Continued surveillance will be useful for monitoring PEDV transmission. Differentiation of the ORF3 genes in PEDV field strains can help us to choose an appropriate PEDV vaccine candidate in the future and prevent outbreaks of PED more effectively.

## Introduction

Porcine epidemic diarrhea virus (PEDV) is one of the major enteric pathogens currently threatening the swine population worldwide (1). Clinically, pigs infected with PEDV cause severe enteric diseases with a high mortality rate in suckling piglets, resulting in tremendous economic losses (2–6). The disease is mainly transmitted through feces (7), air (8), and contaminated feeds (9).

PEDV first emerged in Europe in the 1970s and then spread across Europe and into Asia (10). In China, outbreaks of PEDV have been observed on most swine breeding farms since late 2010 (6, 8, 11). PEDV has rapidly spread across 34 states of America, Canada, and has returned to devastate the swine industry in Asia after being diagnosed in the USA in April 2013 (12, 13).

PEDV is an enveloped, positive-stranded RNA virus in the genus *Alphacoronavirus*, family *Coronaviridae*, order *Nidovirales* (14, 15). The genome of a PEDV is ~28 kb in length and is composed of seven open reading frames (ORFs) arranged in the order 5′-ORF1a/1b-S-ORF3-E-M-N-3′, which encodes four structural proteins and 17 non-structural proteins (nsp1-nsp16, and ORF3) (16, 17).

The full length of the ORF3 gene is 675 bp, encoding 224 amino acids. The PEDV ORF3 gene has been found to have a low sequence conservation through analysis of the amino acid sequences and their homologs across the alpha-coronavirus genus (18, 19). The ORF3 encodes an ion channel protein and regulates virus production (20), and its naturally truncated form might cause attenuation of the virus to the natural host. The differentiation of ORF3 could be a marker of adaptation to cell cultures and attenuation of virus, and this could be a valuable tool for studying the molecular epidemiology of PEDV (5, 21). To investigate the genetic diversity of PEDV in Guangxi, we sequenced the full-length ORF3 gene of 33 PEDV positive field samples collected from 2017 to 2019.

## Materials and Methods

Thirty-three intestinal samples were taken from piglets with clinical diarrhea from different pig farms in Guangxi between 2017 and 2019. Thirty-eight strains of PEDV ORF3 were obtained. Five strains were clustered into a new group referred to as Group 3, while there were 24 and nine strains which were clustered into the G1-1 and G1-3 subgroups, respectively.

Samples were homogenized with a 20% glycerin and PBS stock preservation solution (GPSs). The suspensions were then vortexed and centrifuged for 5 min at 3,000 × g. The supernatants were collected and stored at −80°C before utilization. The vaccine strains CV777 (Harbin Weike Biological Co. Ltd, Harbin, China), AJ1102-R (Wuhan Keqian Biological Co., Ltd, Wuhan, China) and Zhejiang-08 (China Animal Husbandry Industry Co., Ltd, Beijing, China) were purchased as controls.

Total RNA was extracted using a humoral virus DNA/RNA kit (Axygen Scientific, Union City, CA, USA), and transcribed into cDNA by Oligo dTs, dNTP mix, and M-MLV Reverse Transcriptase reagent (TaKaRa, Dalian, China). The primers, ORF3F: 5′-GTCCTAGACTTCAACCTTACGAAG-3′ and ORF3R: 5′-AACTACTAGACCATTATCATTCAC-3′ were used for PCR at 95°C for 5 min followed by 30 cycles of denaturation at 95 °C for 15 s, annealing at 55°C for 30 s and extension at 72°C for 1 min (22). The expected size of PCR products is 740 bp, which contained the full ORF3 gene with a length of 675 bp.

The RT-PCR products were analyzed using 1.5% agarose gel electrophoresis and visualized by ultraviolet illumination. The expected DNA band was purified using a Gel Extraction Kit (OMEGA biotech, Doraville, GA, USA), and cloned into a pMD-18T vector (TaKaRa, Dalian, China). The sequences of the positive clones were determined by Beijing Genomics Institute (Guangzhou, China). The validated genome sequences of ORF3 were submitted to GenBank under the accession numbers MK895557~MK895560 and MN518432~MN518465.

One-hundred and thirteen reference strains of PEDV ORF3 from different countries collected on different dates were selected for the genetic analysis (Supplementary Table 1). Sequences were analyzed by using software packages, DNAstar, MEGA5.2, and iTOL v.5. Multiple nucleotide and amino acid sequence alignments were analyzed by applying the ClustalV method with the MegAlign program and the ClustalW alignment tool in the MEGA5.2 software, respectively. The MEGA 5.2 program was applied to construct phylogenetic trees by using the neighbor-joining method, the tree topology was constructed using the Poisson model and the robustness of the phylogenetic tree was evaluated by bootstrapping using 1,000 replicates (23). The resulting tree was visualized by using iTOL v.5 (Interactive Tree of Life, http://itol.embl.de/).

## Results

### Amplification and Identification of the PEDV ORF3 Gene

**Figure 1:**
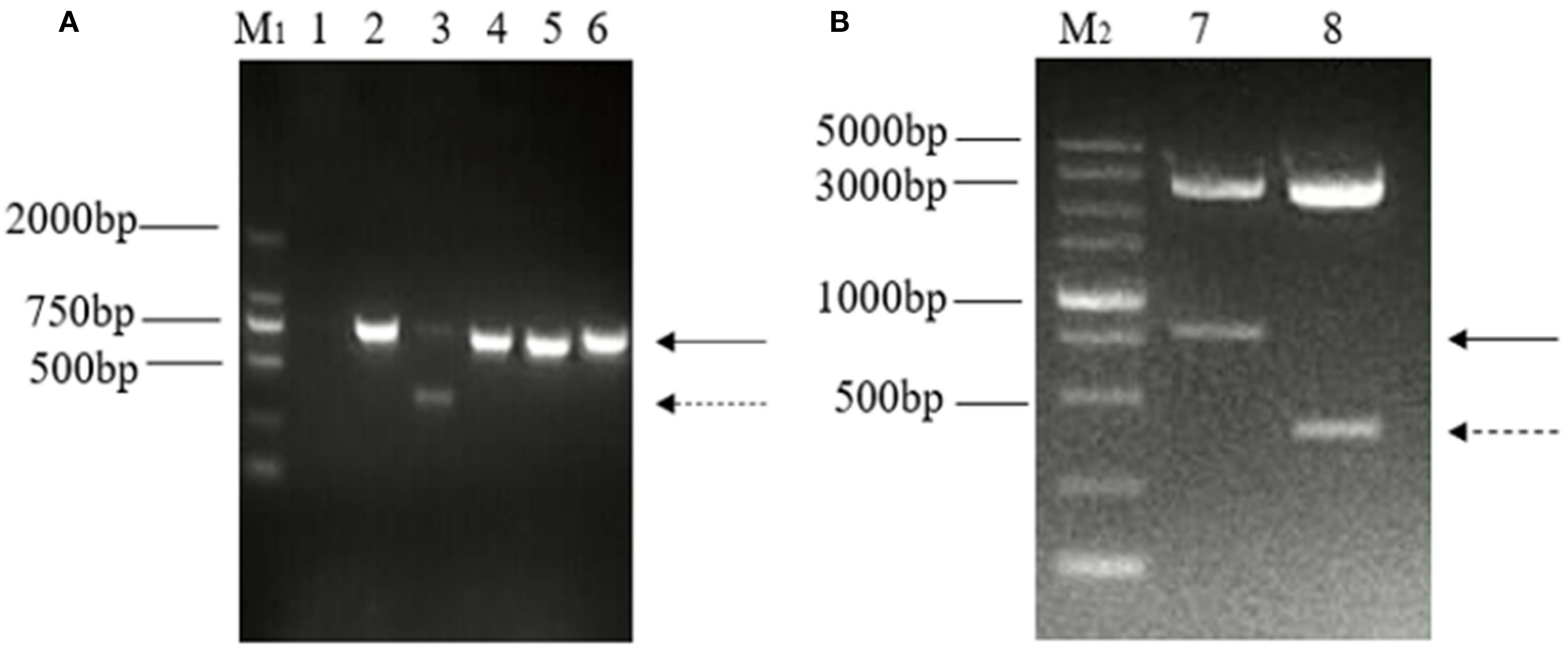
Detection and identification of two distinct bands of the ORF3 gene of PEDV in Guangxi. **(A)** Amplification of ORF3 gene in PEDV positive samples. M_1_: DL 2000 marker; Lane 1: negative control; Lane 2: predicted product (740 bp); Lane 3: predicted product (740 bp) and large genomic deletion (~358 bp); Lane 4: CV777 strain; Lane 5: Zhejiang-08 strain; Lane 6: AJ1102 strain. **(B)** Identification of the plasmid, pMD-18T-ORF3, by enzyme digestion using PstI and BamHI. M_2_: DL 5000 marker; Lane 1: The solid arrows indicate the predicted products (740 bp); Lane 2: The dashed arrows indicate products of PEDV variants with a large genomic deletion (~358 bp).

The ORF3 gene was amplified using the primer pair for ORF3 from generated cDNA, and one distinct band of unexpected shorter sizes was observed in five PCR products in addition to the predicted band. Both bands of nucleotide were cloned into a pMD-18T vector and were identified by enzyme digestion using *PstI* and *BamHI* (Figure 1).

### Nucleotide Alignment of the ORF3 Gene Sequence

**Figure 2:**
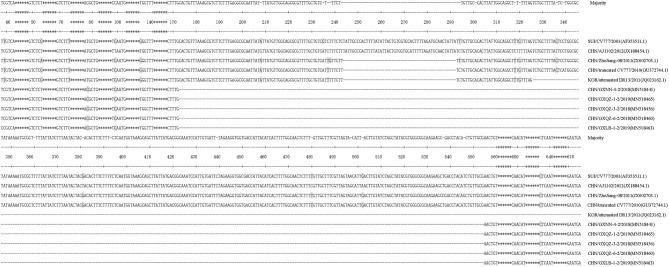
Alignment of nucleotide sequences of ORF3 genes of the Guangxi naturally truncated strains and reference strains. Multiple nucleotide sequence alignments were analyzed by applying the Clustal V method with the MegAlign program. The asterisks indicate the genes with no differences found and those not shown in the figure, whereas the dashed lines represent the deleted nucleotides, and the shadows indicate the unique substitutions of the Guangxi naturally truncated strains.

In this study, thirty-eight strains of PEDV ORF3 were obtained from 33 samples in Guangxi between 2017 and 2019 (Table 1). There were five strains (CHN/GXNN-4-2/2018, CHN/GXQZ-3-2/2018, CHN/GXQZ-6-2/2018, CHN/GXLB-1-2/2019 and CHN/GXQZ-1-2/2019) of ORF3 which were only 293bp in length, and the rest of the 33 strains had a complete ORF3 gene sequence, with a length of 675 bp. When these five Guangxi naturally truncated strains were compared with the main reference strains for nucleotide alignment (Supplementary Table 1), we found that the five strains all had continuous deletions from 172 to 554 bp, and exhibited 98.0–99.3% nucleotide identity with them. In addition, the Guangxi naturally truncated strains had the highest homology of up to 95.9–96.6%, when compared with AJ1102, and exhibited 94.2–95.6% nucleotide identity when compared with the CV777, truncated CV777 strain, Zhejiang-08 and attenuated DR13. Interestingly, one unique substitution at C78T was found to be present in the Guangxi naturally truncated strains in the present study (Figure 2), as well as two unique substitutions at T99C and T636C in the AJ1102 strain.

**Table 1:** Origins and information regarding the Guangxi strains from 2017 to 2019.

**Name**	**Collection area**	**Collection date**	**ORF3 gene size**	**Accession number**
CHN/GXWZ-1/2017	Wuzhou	2017.08	675 bp	MN518432
CHN/GXWZ-2/2017	Wuzhou	2017.08	675 bp	MN518433
CHN/GXNN-1/2017	Nanning	2017.11	675 bp	MK895557
CHN/GXQZ-1/2017	Qinzhou	2017.11	675 bp	MN518434
CHN/GXGG-1/2018	Guigang	2018.03	675 bp	MN518435
CHN/GXYL-1/2018	Yulin	2018.03	675 bp	MN518452
CHN/GXNN-5/2018	Nanning	2018.03	675 bp	MN518461
CHN/GXLZ-1/2018	Liuzhou	2018.04	675 bp	MN518449
CHN/GXBH-1/2018	Beihai	2018.05	675 bp	MN518442
CHN/GXNN-1/2018	Nanning	2018.06	675 bp	MK895558
CHN/GXQZ-1/2018	Qinzhou	2018.07	675 bp	MN518453
CHN/GXQZ-2/2018	Qinzhou	2018.08	675 bp	MN518454
CHN/GXQZ-3-1/2018	Qinzhou	2018.08	675 bp	MN518455
CHN/GXQZ-3-2/2018	Qinzhou	2018.08	293 bp	MN518456
CHN/GXQZ-4/2018	Qinzhou	2018.08	675 bp	MN518457
CHN/GXQZ-6-1/2018	Qinzhou	2018.08	675 bp	MN518459
CHN/GXQZ-6-2/2018	Qinzhou	2018.08	293 bp	MN518460
CHN/GXGG-2/2018	Guigang	2018.10	675 bp	MN518436
CHN/GXNN-2/2018	Nanning	2018.10	675 bp	MK895559
CHN/GXBH-2/2018	Beihai	2018.10	675 bp	MN518443
CHN/GXBH-6/2018	Beihai	2018.10	675 bp	MN518447
CHN/GXBH-7/2018	Beihai	2018.10	675 bp	MN518448
CHN/GXQZ-5/2018	Qinzhou	2018.10	675 bp	MN518458
CHN/GXNN-4-1/2018	Nanning	2018.11	675 bp	MN518440
CHN/GXNN-4-2/2018	Nanning	2018.11	293 bp	MN518441
CHN/GXBH-3/2018	Beihai	2018.11	675 bp	MN518444
CHN/GXBH-4/2018	Beihai	2018.11	675 bp	MN518445
CHN/GXBH-5/2018	Beihai	2018.11	675 bp	MN518446
CHN/GXLB-1/2018	Laibin	2018.11	675 bp	MN518450
CHN/GXLB-2/2018	Laibin	2018.11	675 bp	MN518451
CHN/GXGG-3/2018	Guigang	2018.12	675 bp	MN518437
CHN/GXNN-3/2018	Nanning	2018.12	675 bp	MK895560
CHN/GXLB-1-1/2019	Laibin	2019.01	675 bp	MN518462
CHN/GXLB-1-2/2019	Laibin	2019.01	293 bp	MN518463
CHN/GXQZ-1-1/2019	Qinzhou	2019.01	675 bp	MN518464
CHN/GXQZ-1-2/2019	Qinzhou	2019.01	293 bp	MN518465
CHN/GXNN-1/2019	Nanning	2019.04	675 bp	MN518438
CHN/CXNN-2/2019	Nanning	2019.04	675 bp	MN518439

**Figure 3:**
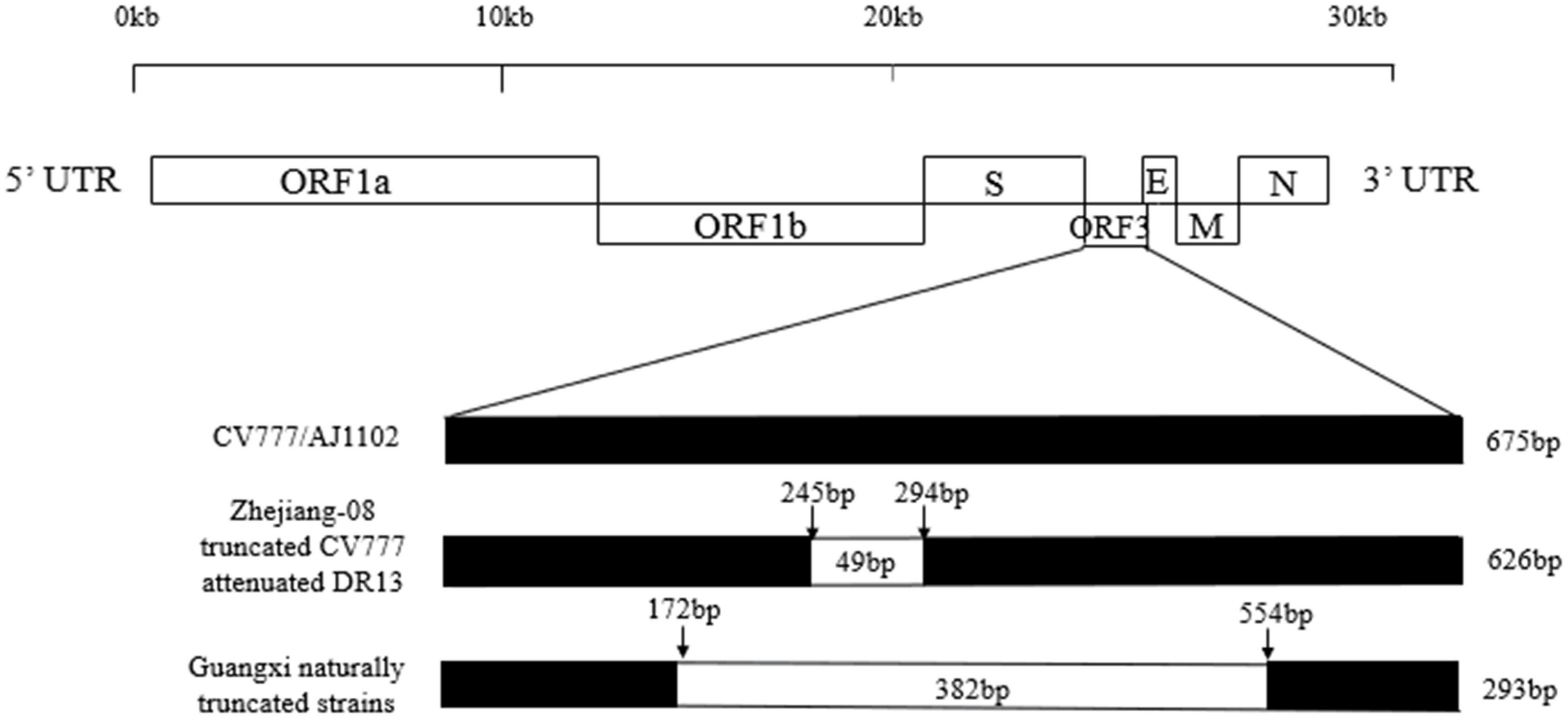
Comparison map of the ORF3 gene deletion in the Guangxi naturally truncated and reference strains. The ORF3 nucleotide sequences of the vaccine strains, CV777 and AJ1102, were 675bp, while the strains of truncated CV777, Zhejiang-08 and attenuated DR13 were 626 bp. The ORF3 of the Guangxi naturally truncated strains were all 293 bp, which contained the deletion area of the reference vaccine strain described above.

A sketch map of the ORF3 comparisons of the strains, including CV777, AJ1102, truncated CV777, Zhejiang-08, attenuated DR13, and the Guangxi naturally truncated strains is shown in Figure 3. The results show that the ORF3 gene of CV777 and AJ1102 were complete, and the truncated CV777, Zhejiang-08 strain and attenuated DR13 strain had 49 nucleotide deletions at 245–294 bp. Importantly, the Guangxi naturally truncated strains contained all the missing regions of these reference strains.

### Alignment of Amino Acid Sequences

**Figure 4:**
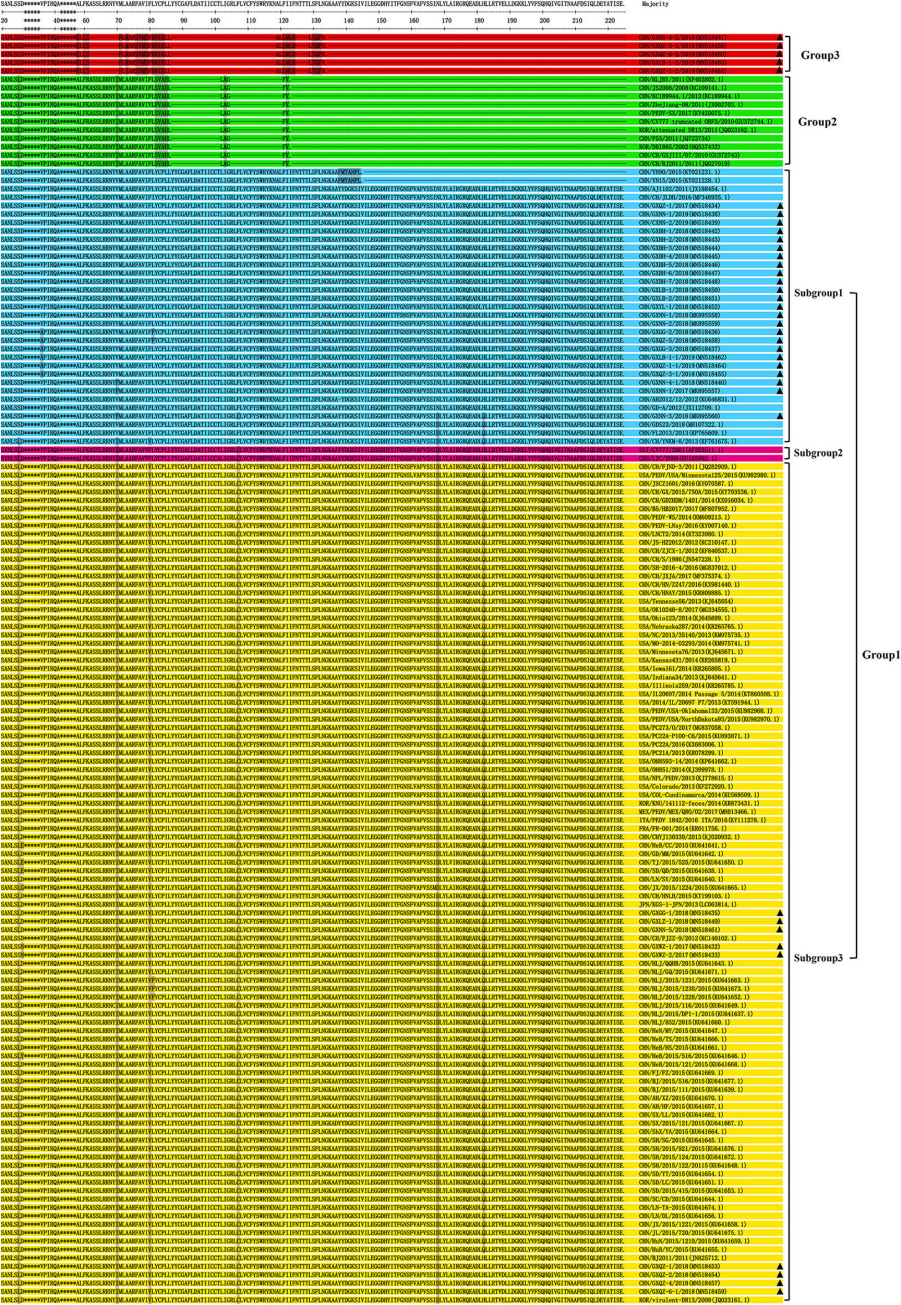
Alignment of amino acid sequences of ORF3 proteins of the Guangxi PEDV and reference strains. The asterisks indicate the segments with no differences and not shown in the figure, whereas the dashed lines indicate deleted amino acids, and the shadows represent the unique substitutions of the chosen strains. Each PEDV strain is indicated in the following format: Country origin (three letter code: CHN, China; FRA, France; ITA, Italy; JPN, Japan; KOR, Korea; MEX, Mexico; SUI, Switzerland; USA, the United States)/strain name/year of sample collection (Genbank accession number). The Group 3 consisted of Guangxi naturally truncated strains which were coded in red. G1-1 subgroup, G1-2 subgroup, G1-3 subgroup and Group 2 were coded in blue, pink, yellow, and green, respectively. The triangle symbols represent the Guangxi field strains obtained in this study.

The results of amino acid sequence analysis indicated that all the strains were separated into three groups (Figure 4). Among the Guangxi strains in this study, 33 strains were 675 bp in length and encoded a protein of 224 amino acids, and these belong to Group 1. The Guangxi naturally truncated strains were 293 bp in length and encoded a truncated protein of 89 amino acids, and these formed a new group referred to as Group 3. The ORF3 genes of the Guangxi naturally truncated strains exhibited 94.4–97.8% amino acids identity, whereas they had 69.3–72.7% identity to the truncated CV777, Zhejiang-08 and attenuated DR13 strains, and 65.2–71.9% identity to the CV777 and AJ1102. Within Group 2, there are 11 reference strains (including truncated CV777, Zhejiang-08, and attenuated DR13 strain) and translation in these strains was terminated early because of specific deletions, which were located at amino acids 86L−102L and 104G−121F, respectively.

In terms of predicted amino acid sequence, strains in G1-1 and G1-3 subgroups all had an ORF3 of 224 amino acids, but their genotypes were different because of the amino acid mutations (5, 24); six specific amino acids (L24S, I70V, V80F, C107F, D168N, and Q182H) were present the G1-3 subgroups (Figure 4). Two strains (CV777 and LZC) were classified as G1-2 subgroups since there were only four substitutions, without V80F and Q182H. The Guangxi naturally truncated strains with 89aa in Group 3 were found to have a long length deletion, which were located at amino acids 61S−71F, 86L−119A, and 124H−129L, respectively. In addition, the L81 is located immediately before the truncation site in Group 2, while Group 2 consisted mostly of the cell-adapted strains.

### Phylogenetic Tree of the Amino Acid Sequences

**Figure 5:**
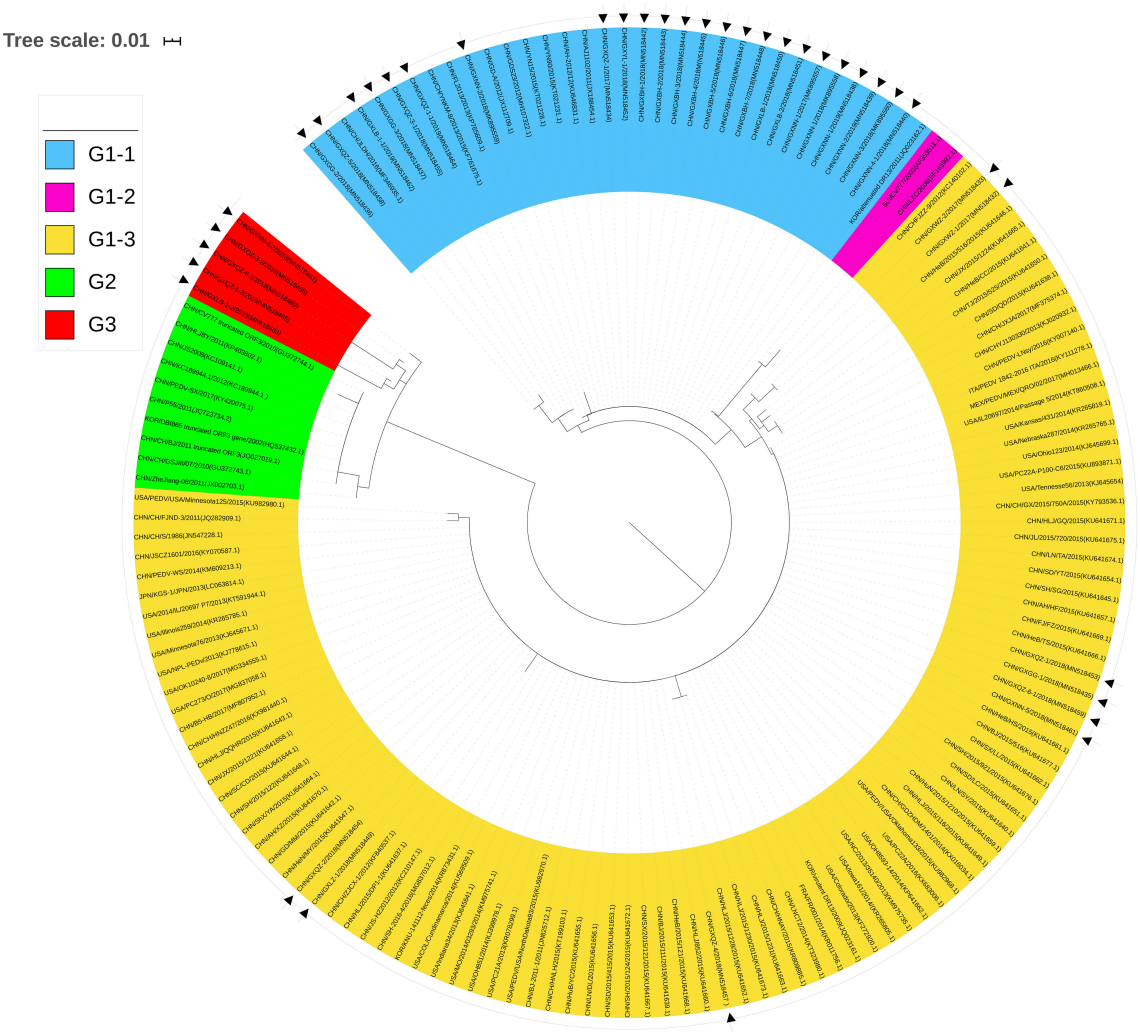
Phylogenetic tree of ORF3 of PEDV samples based on the amino acid sequences. Sequences of reference and vaccine strains were obtained from GeneBank, the accession numbers are shown in Supplementary Table 1. Tree topology was constructed using the Poisson model and bootstrap re-sampling (1,000 data sets) of the multiple alignments was used to test the statistical robustness of the trees obtained by the neighbor-joining method from MEGA 5.2. Each PEDV strain is indicated in the following format: Country origin (three letter code: CHN, China; FRA, France; ITA, Italy; JPN, Japan; KOR, Korea; MEX, Mexico; SUI, Switzerland; USA, the United States)/strain name/year of sample collection (Genbank accession number). The Group 3 consisted of Guangxi naturally truncated strains which were coded in red. G1-1 subgroup, G1-2 subgroup, G1-3 subgroup, and Group 2 were coded in blue, pink, yellow, and green, respectively. The triangle symbols represent the Guangxi field strains obtained in this study.

To analyze the phylogenetic relationships of the 38 strains (Table 1) and the 113 reference strains from various parts of the world (Supplementary Table 1), we constructed a neighbor-joining phylogenetic tree using their ORF3 amino acid sequences (Figure 5).

Twenty-four strains were clustered into the G1-1 subgroup together with the major domestic strains such as YN15 and vaccine strains AJ1102 and GD-A, while nine strains were clustered to the G1-3 subgroup with major foreign strains such as PC22A and OH851. CV777 and LZC strains were clustered into the G1-2 subgroup. The deletions of the ORF3 gene such as the truncated CV777, Zhejiang-08 and other strains were clustered into Group 2. More importantly, the Guangxi naturally truncated strains were clustered into a new group referred to as Group 3.

## Discussion

PED is a highly contagious intestinal infectious disease in pigs caused by PEDV, which has seriously affected the development of the pig breeding industry and caused significant economic losses. Since 2010, there has been a new round of PED epidemic in China (11, 21, 25, 26). In addition, most pig production countries in Europe, America, and Southeast Asia also have a large-scale epidemic of PED (27–29). It has recently been proposed that PEDV ORF3 plays a role in regulating PEDV replication and pathogenesis (1, 20, 30). Moreover, it has been demonstrated, by using reverse genetics systems, that the ORF3 is dispensable for viral growth *in vitro* (31–33). The 49 nucleotide deletion at 245–294 bp is thought to be a marker of cellular adaptation, because most cell-adapted strains contain these deletions, but the evaluation of ORF3 slightly changed strains on cell culture need to be further investigated. It can also be used to distinguish the cell adaptive strains from the wild strains (34). This suggests that ORF3 may be a multifunctional protein involved in cellular processes, but the exact biological function of PEDV ORF3 needs to be further defined (35). Therefore, understanding the genetic variations of the ORF3 gene is crucial for further studies on the biological functions of PEDV.

In this study, 38 PEDV strains from Guangxi were obtained from 33 field samples collected on pig farms. Among them, 24 strains were clustered into the G1-1 subgroup, and these were in the same branch as the reference strains, such as vaccine strain AJ1102, and 9 strains were in a branch of the G1-3 subgroup with reference strains such as foreign strains PC22A and OH851. Importantly, there were 5 strains with naturally truncated ORF3 genes and these formed a new gene group in a separate branch, and these were different when compared to the highly adapted cell strains such as the Chinese HLJBY and JS2008. We showed that the length of the deduced amino acid sequences of the ORF3 had only 89aa which was unique among the tested strains. Based on the genetic and evolutionary analysis of the ORF3 gene, we found that the endemic PEDV strains in Guangxi were complicated with three genotypes (G1-1 subgroup, G1-3 subgroup, and Group 3) co-existing simultaneously. The S protein is known to be an appropriate viral gene for determining the genetic relatedness among PEDV isolates, and the non-S INDEL and classical genogroups can be clearly differentiated in an S protein phylogenetic tree (7, 36). The variants in the S protein may change the virulence and tissue tropism of PEDV. However, the ORF3 and S gene are under different evolutionary pressures, and the correlation between the mutation rate of ORF3 gene and the pressure of selection of the host's immune response need to be further investigated.

There has been at least one type of ORF3 gene deletion reported. It is the type of deletion which mostly occurs during extensive passaging of the virus in cell cultures and this has been correlated with viral attenuation (19). With the deletion, the ORF3 gene of an attenuated-type virus has 49 nucleotide deletions, and this leads to a reading frame-shift and an early termination of translation (20). It is mainly represented by the strains of truncated CV777, Zhejiang-08, JS2008, HLJBY, and attenuated DR13, which only has a 91aa truncated protein (18, 24, 37–39). It is noteworthy that the ORF3 protein of the Guangxi naturally truncated strains contain only residues of 89aa, since the 382-nucleotide deletion in the ORF3 results in a frameshift mutation and an early termination of translation. Like the L81 which is critical for PEDV rescue in reverse genetics and could possibly play an important role in the inhibitory activity of the ORF3 (30), the amino acid site of the E58 is located immediately before the truncation site in the five Guangxi natural truncated strains, and this specific region is due to the mutation of the amino acids, but the function of this is unknown. The effect of the Guangxi naturally truncated strains on ORF3 functions and viral pathogenicity remains to be investigated. It is worth considering whether the truncated part of the ORF3 gene should be used as a genetic marker for establishing methods to distinguish between different strains.

PEDV strains with naturally occurring truncated ORF3 genes were found from different parts of Guangxi, originating from Qinzhou, Nanning, and Laibin, which suggested that this type of PEDV is present in only certain areas. Interestingly, the Guangxi naturally truncated strains all co-exist with strains of the G1-1 subgroup which contain complete ORF3 genes, which means that this type of PEDV may not infect pigs independently. The presence of different subtypes of PEDV in pigs may accelerate the mutation of the virus, and this can also increase the difficulty for the clinical prevention and control of PEDV. We have purified these two types of viruses by using the plaque assay and pathogenicity studies are currently in progress with these. The effect of the deletion of ORF3 associated with viral replication or virulence would be important for the understanding the gene function. Previously, JS2008 was considered to be a recombination of a vaccine strain and a PEDV variant strain (38). It is not possible to speculate whether these five strains with naturally truncated ORF3 genes is a recombination of the vaccine strain and a PEDV variant strain, and this needs further confirmation.

This study identified 38 strains of PEDV ORF3 in the Guangxi province of China from 2017 to 2019. There were five naturally truncated strains which were clustered into a new group and these had longer length deletions in both nucleotides and amino acids sequences. This will hopefully facilitate the establishment of a diagnostic method that can differentiate the PEDV field stains. Further studies are needed to clarify the effects of the naturally truncated ORF3 gene on the virulence of these PEDV strains. Phylogenetic analysis revealed that three types PEDV strains, G1-1 subgroup, G1-3 subgroup and Group 3, co-circulated in the swine population in the Guangxi province of China. Continued surveillance will be useful for monitoring PEDV transmission. Differentiation of ORF3 genes in PEDV field strains can also help us to choose an appropriate PEDV vaccine candidate in the future and prevent outbreaks of PED more effectively.

## Data Availability Statement

The datasets generated for this study can be found in the GenBank MK895557~MK895560 and MN518432~MN518465.

## Ethics Statement

The animal study was reviewed and approved by the Animal Care & Welfare Committee of Guangxi University.

## Author Contributions

WH, YLi, and KO: conceptualization and project administration. YLu, LM, PK, RW, LZ, CY, YC, and ZW: data curation. YLu, XS, CD, and YC: formal analysis. WH and KO: funding acquisition, supervision, and writing—review and editing. CY and YLi: resources. YLu, XS, and CD: writing—original draft.

## Conflict of Interest

The authors declare that the research was conducted in the absence of any commercial or financial relationships that could be construed as a potential conflict of interest.
