# Supplementary material for: Genetic Diversity of Porcine Epidemic Diarrhea Virus With a Naturally Occurring Truncated ORF3 Gene Found in Guangxi, China
Source: Front Vet Sci. 2020 Jul 24;7:435. doi: 10.3389/fvets.2020.00435 (PMC7393948; doi:10.3389/fvets.2020.00435)
Supplement: Supplementary file 1 [file Table_1.DOCX]

Supplementary Material

# Supplementary Tables 1. Information regarding the reference strains.

| **Virus strain** | **Country** | **Collected date** | **Accession number** |
| --- | --- | --- | --- |
| CH/S | China | 1986 | JN547228.1 |
| CV777 | Switzerland | 2001 | AF353511.1 |
| OH851 | USA | 2001 | KJ399978.1 |
| DBI865 truncated ORF3 | South Korea | 2002 | HQ537432.1 |
| LZC | China | 2006 | EF185992.1 |
| JS2008 | China | 2008 | KC109141.1 |
| virulent DR13 | South Korea | 2009 | JQ023161.1 |
| CV777 truncated ORF3 | China | 2010 | GU372744.1 |
| CH/GSJIII/07 | China | 2010 | GU372743.1 |
| CH/FJND-3/2011 | China | 2011 | JQ282909.1 |
| ZheJiang-08 | China | 2011 | JX002703.1 |
| AJ1102 | China | 2011 | JX188454.1 |
| BJ-2011-1 | China | 2011 | JN825712.1 |
| attenuated DR13 | South Korea | 2011 | JQ023162.1 |
| CH/BJ/2011 truncated ORF3 | China | 2011 | JQ027019.1 |
| P55 | China | 2011 | JQ723734.2 |
| AH 2012/12 | China | 2012 | KU646831.1 |
| CH/FJZZ-9/2012 | China | 2012 | KC140102.1 |
| CH/ZJCX-1/2012 | China | 2012 | KF840537.1 |
| GD-A | China | 2012 | JX112709.1 |
| GDS23 | China | 2012 | MH107322.1 |
| JS-HZ2012 | China | 2012 | KC210147.1 |
| KC189944.1 | China | 2012 | KC189944.1 |
| CHYJ130330 | China | 2013 | KJ020932.1 |
| FL2013 | China | 2013 | KP765609.1 |
| KGS-1/JPN/2013 | Japan | 2013 | LC063814.1 |
| NPL-PEDv/2013 | USA | 2013 | KJ778615.1 |
| PC21A | USA | 2013 | KR078299.1 |
| PC22A | USA | 2013 | KX683006.1 |
| USA/Colorado/2013 | USA | 2013 | KF272920.1 |
| USA/Indiana34/2013 | USA | 2013 | KJ645641.1 |
| USA/Minnesota76/2013 | USA | 2013 | KJ645671.1 |
| USA/NC/2013/35140 | USA | 2013 | KM975735.1 |
| USA/Tennesse56/2013 | USA | 2013 | KJ645654.1 |
| CH/GDZHDM/1401 | China | 2014 | KX016034.1 |
| LNCT2 | China | 2014 | KT323980.1 |
| PEDV-WS | China | 2014 | KM609213.1 |
| FR/001/2014 | France | 2014 | KR011756.1 |
| KNU-141112-feces | South Korea | 2014 | KR873431.1 |
| COL/Cundinamarca/2014 | USA | 2014 | KU569509.1 |
| OH8593-14 | USA | 2014 | KP641662.1 |
| USA/2014/IL/20697 P7 | USA | 2014 | KT591944.1 |
| USA/IL20697/2014 Passage 5 | USA | 2014 | KT860508.1 |
| USA/Illinois259/2014 | USA | 2014 | KR265785.1 |
| USA/Iowa161/2014 | USA | 2014 | KR265805.1 |
| USA/Kansas431/2014 | USA | 2014 | KR265819.1 |
| USA/MO/2014/03293 | USA | 2014 | KM975741.1 |
| USA/Nebraska287/2014 | USA | 2014 | KR265765.1 |
| USA/Ohio123/2014 | USA | 2014 | KJ645699.1 |
| HLJBY | China | 2015 | KP403802.1 |
| CH/GX/2015/750A | China | 2015 | KY793536.1 |
| CH/HNAY/2015 | China | 2015 | KR809885.1 |
| CH/HNLH/2015 | China | 2015 | KT199103.1 |
| CH/YNKM-8/2013 | China | 2015 | KF761675.1 |
| YN15 | China | 2015 | KT021228.1 |
| YN90 | China | 2015 | KT021231.1 |
| PC22A-P100-C6 | USA | 2015 | KU893871.1 |
| PEDV/USA/Minnesota125/2015 | USA | 2015 | KU982980.1 |
| PEDV/USA/NorthDakota93/2015 | USA | 2015 | KU982970.1 |
| PEDV/USA/Oklahoma133/2015 | USA | 2015 | KU982968.1 |
| HLJ2015/DP1-1 | China | 2015 | KU641637.1 |
| SD/QD/2015 | China | 2015 | KU641638.1 |
| BJ/2015/111 | China | 2015 | KU641639.1 |
| LN/SY/2015 | China | 2015 | KU641640.1 |
| HeB/CC/2015 | China | 2015 | KU641641.1 |
| GD/MM/2015 | China | 2015 | KU641642.1 |
| HLJ/QQHR/2015 | China | 2015 | KU641643.1 |
| SC/CD/2015 | China | 2015 | KU641644.1 |
| SH/SG/2015 | China | 2015 | KU641645.1 |
| HeB/2015/516 | China | 2015 | KU641646.1 |
| HeN/MY/2015 | China | 2015 | KU641647.1 |
| SH/2015/122 | China | 2015 | KU641648.1 |
| HLJ/2015/116 | China | 2015 | KU641649.1 |
| TJ/2015/525 | China | 2015 | KU641650.1 |
| SD/LC/2015 | China | 2015 | KU641651.1 |
| HLJ/2015/1228 | China | 2015 | KU641652.1 |
| SD/2015/415 | China | 2015 | KU641653.1 |
| SD/YT/2015 | China | 2015 | KU641654.1 |
| HuB/YC/2015 | China | 2015 | KU641655.1 |
| LN/DL/2015 | China | 2015 | KU641656.1 |
| AH/HF/2015 | China | 2015 | KU641657.1 |
| JX/2015/1221 | China | 2015 | KU641658.1 |
| HuN/2015/1210 | China | 2015 | KU641659.1 |
| HLJ/852/2015 | China | 2015 | KU641660.1 |
| HeB/HS/2015 | China | 2015 | KU641661.1 |
| SX/LL/2015 | China | 2015 | KU641662.1 |
| HLJ/2015/1231 | China | 2015 | KU641663.1 |
| ShX/YA/2015 | China | 2015 | KU641664.1 |
| JX/2015/1224 | China | 2015 | KU641665.1 |
| HeB/TS/2015 | China | 2015 | KU641666.1 |
| SX/2015/121 | China | 2015 | KU641667.1 |
| HeB/2015/121 | China | 2015 | KU641668.1 |
| FJ/FZ/2015 | China | 2015 | KU641669.1 |
| AH/XZ/2015 | China | 2015 | KU641670.1 |
| HLJ/GQ/2015 | China | 2015 | KU641671.1 |
| SH/2015/124 | China | 2015 | KU641672.1 |
| HLJ/2015/1230 | China | 2015 | KU641673.1 |
| LN/TA/2015 | China | 2015 | KU641674.1 |
| JL/2015/720 | China | 2015 | KU641675.1 |
| SH/2015/921 | China | 2015 | KU641676.1 |
| BJ/2015/516 | China | 2015 | KU641677.1 |
| CH/JLDH/2016 | China | 2016 | MF346935.1 |
| CH/HNZZ47/2016 | China | 2016 | KX981440.1 |
| CHN/SH-2016-4/2016 | China | 2016 | MG837012.1 |
| JSCZ1601 | China | 2016 | KY070587.1 |
| PEDV-LNsy | China | 2016 | KY007140.1 |
| PEDV 1842/2016 ITA | Italy | 2016 | KY111278.1 |
| B5-HB2017 | China | 2017 | MF807952.1 |
| CH/JXJA/2017 | China | 2017 | MF375374.1 |
| PEDV-SX | China | 2017 | KY420075.1 |
| PEDV/MEX/QRO/02/2017 | Mexico | 2017 | MH013466.1 |
| PC273/O | USA | 2017 | MG837058.1 |
| USA/OK10240-8/2017 | USA | 2017 | MG334555.1 |
